# Complete Genome Sequence of Citrobacter koseri Strain MPUCK001

**DOI:** 10.1128/MRA.01228-20

**Published:** 2020-12-10

**Authors:** Tomotaka Ohkubo, Yasuhiko Matsumoto, Otomi Cho, Yuki Ogasawara, Takashi Sugita

**Affiliations:** a Department of Microbiology, Meiji Pharmaceutical University, Kiyose, Tokyo, Japan; b Department of Analytical Biochemistry, Meiji Pharmaceutical University, Kiyose, Tokyo, Japan; University of Southern California

## Abstract

Citrobacter koseri, an aerobic Gram-negative bacterium, is isolated from the human skin and intestinal tract. Here, we report the complete genome sequence of Citrobacter koseri strain MPUCK001, which has a 4.9-Mbp genome, containing 4,536 protein-coding sequences.

## ANNOUNCEMENT

Citrobacter koseri is a motile bacterium that is oxidase negative and indole positive and uses citrate as a carbon source (1). *C. koseri* is present in the environment, including the soil, and is also isolated from the skin and intestinal tract of humans (2). Clinically, *C. koseri* causes severe meningitis and brain abscesses in neonates and immunocompromised patients (3). Outbreaks due to nosocomial infections have also been reported (4, 5). Elucidating the characteristics of *C. koseri* is an important topic for clinical application.

The *C. koseri* strain MPUCK001 sample was collected from the neck of a 23-year-old male with atopic dermatitis using the swab method (6) (institutional review board approval number 201903). The sample was inoculated into Reasoner's 2A (R2A) broth containing 300 µg/ml vancomycin and 5 μg/ml amphotericin B, with aerobic incubation at 32°C for 48 h to enrich the bacterium. The culture medium (100 µl) was then applied to nutrient agar supplemented with 160 μg/ml ampicillin and aerobically cultured at 32°C. A single colony was isolated and cultured on nutrient agar at 32°C. The *C. koseri* cells were harvested from the cultured nutrient agar plate by suspending them with saline. High-molecular-weight genomic DNA was extracted using the Quick-DNA fungal/bacterial miniprep kit (Zymo Research, Irvine, CA, USA). Long-read and short-read sequencing of the obtained genomic DNA was performed at the Oral Microbiome Center at Taniguchi Dental Clinic in Japan.

For long-read sequencing, a DNA library was prepared using a ligation sequencing kit (LSK109) (Oxford Nanopore Technologies [ONT]) and sequenced with a GridION X5 system (ONT) on an R9.4.1 flow cell (FLO-MIN106 R9.41 flow cell). Raw sequence data were base called using Guppy v.3.6.0 (ONT), and the estimated *N*_50_ was 16.14 kb. After quality trimming (average Phred quality value of >10.0) using NanoFilt v.2.7.1 (7), a total of 104,661 reads (1.2 Gb) were generated. For short-read sequencing, DNBSEQ 2 × 150-bp paired-end sequencing was performed using the DNBSEQ-G400RS FAST sequencing instrument (MGI Tech, Shenzhen, China) according to the manufacturer’s instructions, yielding 3,132,966 paired-end reads. To trim adapters and low-quality data, raw sequencing data were preprocessed by FASTQ v.0.20.1 (8), yielding 2,661,960 (158× coverage) short reads with an average length of 145.9 bp.

A total of 104,661 long reads passing the quality check were used to assemble the genome sequence using Flye v.2.8 (9). The resulting circular genome sequence was polished using Pilon v.1.23 (10). To confirm the absence of structural misassembly of the circular contigs, we used the software program SV-Quest (K. Uesaka, https://github.com/kazumaxneo/SV-Quest), which maps short-read sequences to the chromosome sequence, and detected no signals for structural gaps or other inconsistencies. Default parameters were used for all software unless otherwise specified. The final chromosome sequence was 4,925,206 bp (G+C content, 53.8%), and the final coverage of the genome was 157.4×. The chromosome sequence was annotated using DFAST v.1.2.7.0 (11), which predicted 4,536 coding sequences, as well as 22 rRNA genes and 85 tRNA genes.

### Data availability.

The closed complete chromosomal sequence was deposited at DDBJ/EMBL/GenBank under the accession number AP023452. Raw sequencing data were deposited in the DDBJ SRA database under the accession numbers DRR243767 (DNBSEQ) and DRR243768 (ONT), respectively.
